# The effect of automated text messaging and goal setting on pedometer adherence and physical activity in patients with diabetes: A randomized controlled trial

**DOI:** 10.1371/journal.pone.0195797

**Published:** 2018-05-02

**Authors:** Linnea A. Polgreen, Christopher Anthony, Lucas Carr, Jacob E. Simmering, Nicholas J. Evans, Eric D. Foster, Alberto M. Segre, James F. Cremer, Philip M. Polgreen

**Affiliations:** 1 Department of Pharmacy Practice and Science, University of Iowa, Iowa City, Iowa, United States of America; 2 Department of Orthopedic Surgery, University of Iowa, Iowa City, Iowa, United States of America; 3 Department of Health and Human Physiology, University of Iowa, Iowa City, Iowa, United States of America; 4 Signal Center for Healthcare Innovation, University of Iowa Health Systems, Iowa City, Iowa, United States of America; 5 Department of Biostatistics, University of Iowa, Iowa City, Iowa, United States of America; 6 Department of Computer Science, University of Iowa, Iowa City, Iowa, United States of America; 7 Departments of Internal Medicine and Epidemiology, University of Iowa, Iowa City, Iowa, United States of America; University of Tennessee Health Science Center, UNITED STATES

## Abstract

**Introduction:**

Activity-monitoring devices may increase activity, but their effectiveness in sedentary, diseased, and less-motivated populations is unknown.

**Methods:**

Subjects with diabetes or pre-diabetes were given a Fitbit and randomized into three groups: Fitbit only, Fitbit with reminders, and Fitbit with both reminders and goal setting. Subjects in the reminders group were sent text-message reminders to wear their Fitbit. The goal-setting group was sent a daily text message asking for a step goal. All subjects had three in-person visits (baseline, 3 and 6 months). We modelled daily steps and goal setting using linear mixed-effects models.

**Results:**

138 subjects participated with 48 in the Fitbit-only, 44 in the reminders, and 46 in the goal-setting groups. Daily steps decreased for all groups during the study. Average daily steps were 7123, 6906, and 6854 for the Fitbit-only, the goal-setting, and the reminders groups, respectively. The reminders group was 17.2 percentage points more likely to wear their Fitbit than the Fitbit-only group. Setting a goal was associated with a significant increase of 791 daily steps, but setting more goals did not lead to step increases.

**Conclusion:**

In a population of patients with diabetes or pre-diabetes, individualized reminders to wear their Fitbit and elicit personal step goals did not lead to increases in daily steps, although daily steps were higher on days when goals were set. Our intervention improved engagement and data collection, important goals for activity surveillance. This study demonstrates that new, more-effective interventions for increasing activity in patients with pre-diabetes and diabetes are needed.

## Introduction

Increasing physical activity is a major public health priority. [1] The benefits of increased activity include improved glycemic control, [2–5] improved lipid profiles, decreased adipose tissue, lower blood pressure, [6–8] and decreased mortality. [9] Regular physical activity can also decrease adverse health events in subjects with some cancers, osteoporosis, arthritis, and depression. [10]

One way to increase physical activity is to encourage subjects to walk more. [11]Increasing daily step counts by 2,000–2,500 steps per day can improve health outcomes. [12]To measure changes in physical activity, many activity-monitoring devices have been developed and in some cases, have been shown to have positive beneficial effects. [13–15] One device that has become popular among consumers and researchers is the Fitbit, a wearable, battery-powered, triaxial accelerometer that uses a set of algorithms to derive step counts from raw accelerometry data. [16] Fitbit has a dedicated website that allows users to track their physical activity over time.

While Fitbits and other such devices may be effective for increasing daily steps in the short-term for already-motivated consumers, their effectiveness in sedentary, diseased, and less motivated populations is not as well understood. Importantly, many users often forget to wear their monitor [17] or lose interest in wearing their monitor over time. In fact, one-third of U.S. consumers who have owned an activity monitor stopped using the device within six months of receiving it. [17] To address these barriers, our team developed a bi-directional, automated text-messaging tool that interacts with Fitbits to promote increased monitor use and overall physical activity. This platform holds great potential for advancing the field as it opens the door for developing low-cost, scalable, yet personalized physical activity behavior change interventions. The purposes of this study were to determine whether automatic text-message reminders would improve Fitbit adherence and/or increase physical-activity levels long-term; and whether regular text-message reminders plus goal setting would improve Fitbit adherence and/or increase physical-activity levels.

## Materials and methods

This was a three-arm randomized controlled trial. We recruited adult subjects ages 19–75, who were obese (BMI>30), had a fasting glucose of 100 or higher in the last year, or who had been diagnosed with type II diabetes but were not currently taking insulin. Subjects were also required to have access to the Internet through a computer or smartphone. We excluded subjects with active or acute mental health problems, significant cognitive impairment, lack of fluency in speaking or understanding English, pregnancy, or contraindications to physical activity. Subjects were recruited via a mass email sent to all University of Iowa students, faculty, staff and retirees and in “The Noon News” a newsletter distributed in all hospital cafeterias daily. Recruitment started on March 25, 2014 and ended on January 16, 2015. We scheduled appointments for eligible subjects who responded to our advertisement. Subjects were consented and interviewed by a specifically trained research assistant in the Clinical Research Unit at the University of Iowa Hospitals and Clinics. Subjects signed written consent forms. We generated 150, 3-digit, random numbers without replacement. When an eligible subject scheduled an appointment, they were given the next available random number (i). Group assignment was determined by g = i mod 3. Subjects with g = 0 were assigned to the Fitbit-only group; subjects with g = 1 were assigned to the reminders group; and subjects with g = 2 were assigned to the goal-setting group.

All subjects were given a Fitbit Zip, which is a small (35.6 x 28.9 x 9.6 mm), wearable, triaxial accelerometer. The Fitbit Zip has been demonstrated as a valid measure of energy expenditure (10.1% mean absolute percent error compared to indirect calorimetry).[18] The Fitbit measures steps on a minute-by-minute frequency for 4–6 months on a single battery, and the data collected are automatically uploaded to the Fitbit website using either a small antenna plugged into a USB port on the subject’s computer or a Bluetooth connection with an app on the subject’s smartphone. The Fitbit website provides users with activity summaries and retains historical information on physical activity. Subjects were instructed to wear their Fitbits each day for 6 months. Also, subjects were given a 40-page brochure about healthy weight loss from the National Institutes of Health. [19]

We obtained data collected by each subject’s Fitbit device via the Fitbit application programming interface (API). Once the data are uploaded to the Fitbit servers, they are accessible (after authentication) via the Fitbit API. Our bi-directional text messaging tool accesses and uses these data in the messages it sends to subjects (specifically how many steps were taken on the previous day). The system is implemented in Python using the Django web framework. Text messages are sent by the server via a commercial web-to-SMS gateway [www.twilio.com]; responses are routed back to the server the same way. Responses are time-stamped upon receipt and automatically inserted into a database.

Subjects were randomized into three groups: (i) Fitbit only, (ii) Fitbit with reminders, and (iii) Fitbit with both reminders and goal setting. The Fitbit-only group served as the control group and was not given any extra information or sent any text messages. Thus, the control group received a Fitbit just as they would from a commercial vendor. Subjects in the reminders group were sent a single daily text message reminding them to wear and sync their Fitbit if they had not worn their monitor the previous day. Non-wear was defined as 0 steps recorded for the entire day. (Fitbit does not distinguish between taking no steps and non-wear.) The goal-setting group received daily goal-setting text messages. All goal-setting subjects were sent a morning text message regarding the previous day’s activity and were asked to set a step goal for the current day. If the subject did not wear their Fitbit, the following message was sent: “Remember to wear your Fitbit! What is your goal for today?” Subjects responded with the number of steps they planned to take. For subjects who wore their Fitbit the previous day, our automated system sent personalized feedback: “Yesterday you achieved 5,934 steps; your goal was 6000 steps. What is your goal for today?” Subjects chose to receive messages at 7:00, 8:00 or 9:00 a.m. For both the reminder and goal-setting groups, the text messages continued for all 180 days of the study.

All groups were also scheduled for 3 in-person testing visits (baseline, 3 months and 6 months). During the baseline visit we measured weight to the nearest 0.5 kg and height to the nearest 0.5 cm (for BMI calculation) using a medical grade scale and stadiometer. Fasting glucose and fasting insulin were assessed from a 5 ml blood draw taken after an 8-hour fast. Resting blood pressure was measured using a professional automated sphygmomanometer and standard guidelines. We calculated the quantitative insulin sensitivity check index (QUICKI) from the insulin and glucose values. [20] During the 3-month visit, we repeated the weight and blood pressure measures only. During the 6-month visit we repeated all baseline measures. Subjects were compensated $25 for each of the three visits and an additional $15 if they returned the Fitbit at the end of the study. The study ended on July 23, 2015.

Our sample size calculation was based on the equations in Diggle (2002) for longitudinal data. [21] We defined a minimally clinically significant effect as 1,000 steps/day and prior studies had reported standard deviations of step counts of ~4,000 steps per day for an effect size of approximately 0.25. Since the power calculation in Diggle applies to a two group comparison and we had three groups, we set our alpha value at 0.025. With two comparisons, our familywise alpha should be below 0.05. Daily step counts are generally uncorrelated but we assumed a correlation coefficient of 0.15. We also expected to lose about 25% of days due to non-compliance with wearing the Fitbit. Using a 180 day trial with these assumptions, we would have 80% power to detect an effect size of at least 0.25 with 47 subjects per arm. In anticipation of dropout and larger non-compliance rates, we planned to recruit 50 subjects per arm.

### Endpoints

The primary endpoint for this study was whether the mean number of steps changed between the three groups over the study. Secondary endpoints are whether compliance rates varied between the groups across the study and whether the act of setting a goal increased steps in the goal-setting group on days when goals were set.

### Statistical analysis

To determine if our randomization was adequate, we compared the initial baseline variables (sex, BMI, BP, glucose, insulin) for the three groups. We calculated either percentages for categorical variables or means and standard deviations for continuous variables. Comparisons across treatment groups were performed using chi-squared tests for categorical variables or using ANOVA for continuous variables. In addition, for any variables that were significantly different among the three groups, further pairwise comparisons were performed using two-sample t-tests.

Second, because we have 6 months of minute-by-minute data, rather than doing a before-and-after analysis, we modeled the entire series. We used a linear mixed-effects model to describe the expected daily number of steps taken in each arm. The model included a random intercept by subject to account for between-subject differences and within-subject correlation of observations and fixed effects for the month of the year, the group membership (goal-setting and reminders vs. Fitbit), the number of days since enrollment and the interaction between group membership and the number of days since enrollment.

For our third analysis, we used only the data from the members of the goal-setting group. We explored the effect of goal setting using the days when subjects set goals as the intervention and days when they did not set goals as the control. We fit a similar linear mixed effects model as above and included the number of goals set as a variable in the model.

For each of the linear mixed effects models, we computed bootstrapped confidence intervals with 5000 repetitions for the reported parameters.

One particular issue unique to pedometer studies is missing data and partial data. A subject may wear their Fitbit and be inactive during a day recording few steps or may be active but only partially wear their Fitbit. The problem of removing the days that are only partially recorded is that such days resemble inactive days introducing the potential for selection bias to be introduced into the data by “trimming”. After some consideration of difference rules (e.g., requiring at least one minute of movement in at least 10 hours of a day to count as “worn”), we settled on a relatively simple rule of removing any records with fewer than 20 minutes of activity across an entire day. If a day had no recorded steps or fewer than 20 minutes of activity, it was set to missing and removed from the analysis. This problem could be averted by using newer devices with heart rate monitoring to have an impartial estimate of wear time; however, such devices were not released at the time of this trial.

All analysis was performed using R. This study was approved by the University of Iowa Institutional Review Board (HawkIRB). This study was funded by a Fraternal Order of Eagles Diabetes Research Center Pilot and Feasibility Grant [PMP], the National Institute of Diabetes and Digestive and Kidney Disorders, grant #5R21DK108019 [PMP], The University of Iowa Health Ventures’ Signal Center for Health Innovation [PMP], and the National Heart, Lung and Blood Institute, grant #K25 HL122405 [LAP].

## Results

**Fig 1:**
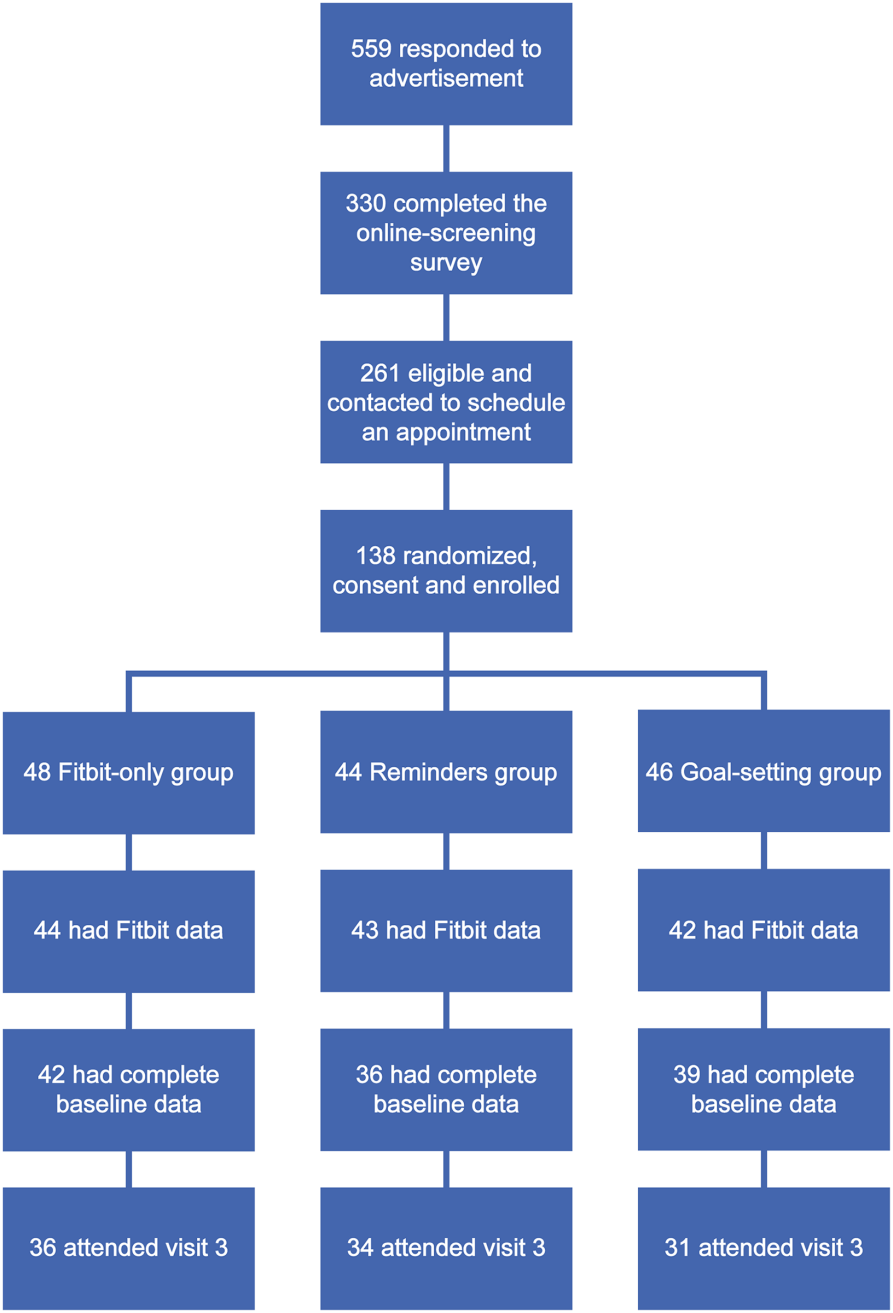
Participant recruitment flow chart.

559 people responded to our request for participants. 330 completed the on-line screening survey, 261 were eligible and were contacted to schedule an appointment. 138 subjects were randomized, consented and enrolled with 48 in the Fitbit-only group, 44 in the reminders group, and 46 in the goal-setting group. (See Fig 1) Baseline demographic data are provided in Table 1. At the α = 0.05 level, there were no statistically significant differences between the study arms in any baseline clinical or demographic characteristics except for diastolic blood pressure (DBP). 37 subjects (26.7%) did not attend visit 3, but these were equally distributed across the three groups: 12 in the Fitbit-only group, 10 in the reminders group, and 15 in the goal-setting group. Of the subjects randomized to groups, 9 failed to wear their Fitbit, and these rates did not vary by arm (p = 0.43).

**Table 1:** Baseline demographic data. Specific sample sizes are displayed where missing values are present. P-values are given for ANOVA tests for continuous variables and chi squared tests for categorical variables.

Variable Name	Group			p-value
	Fitbit only N = 48	Reminders N = 44	Goal setting N = 46	
Gender: Female (%)	35 (74.5%) N = 47	34 (77.3%) N = 44	36 (78.3%) N = 46	0.904
Age (Years)	44.6 (16.7) N = 42	47.4 (15.1) N = 40	43.0 (16.0) N = 38	0.670
Height (cm)	170.4 (8.6)	171.7 (9.3)	168.3 (9.1)	0.199
Weight (kg)	109.6 (19.6)	107.9 (20.1)	107.0 (21.2)	0.823
BMI (kg/m^2^)	37.8 (6.8)	36.5 (5.8)	37.7 (6.6)	0.575
Systolic Blood Pressure (mmHg)	132.0 (13.2)	135.6 (15.8)	137.2 (11.4)	0.162
Diastolic Blood Pressure (mmHg)	75.2 (8.2)	77.2 (9.0)	80.0 (9.3)	0.032
Glucose (mg/dL)	103.5 (19.9) N = 44	116.4 (39.7) N = 37	106.6 (29.4) N = 45	0.145
Insulin	18.5 (13.5) N = 47	19.1 (14.6) N = 43	22.0 (26.2) N = 45	0.642
QUICKI	0.14 (0.01) N = 43	0.14 (0.01) N = 37	0.14 (0.01) N = 44	0.343

**Fig 2:**
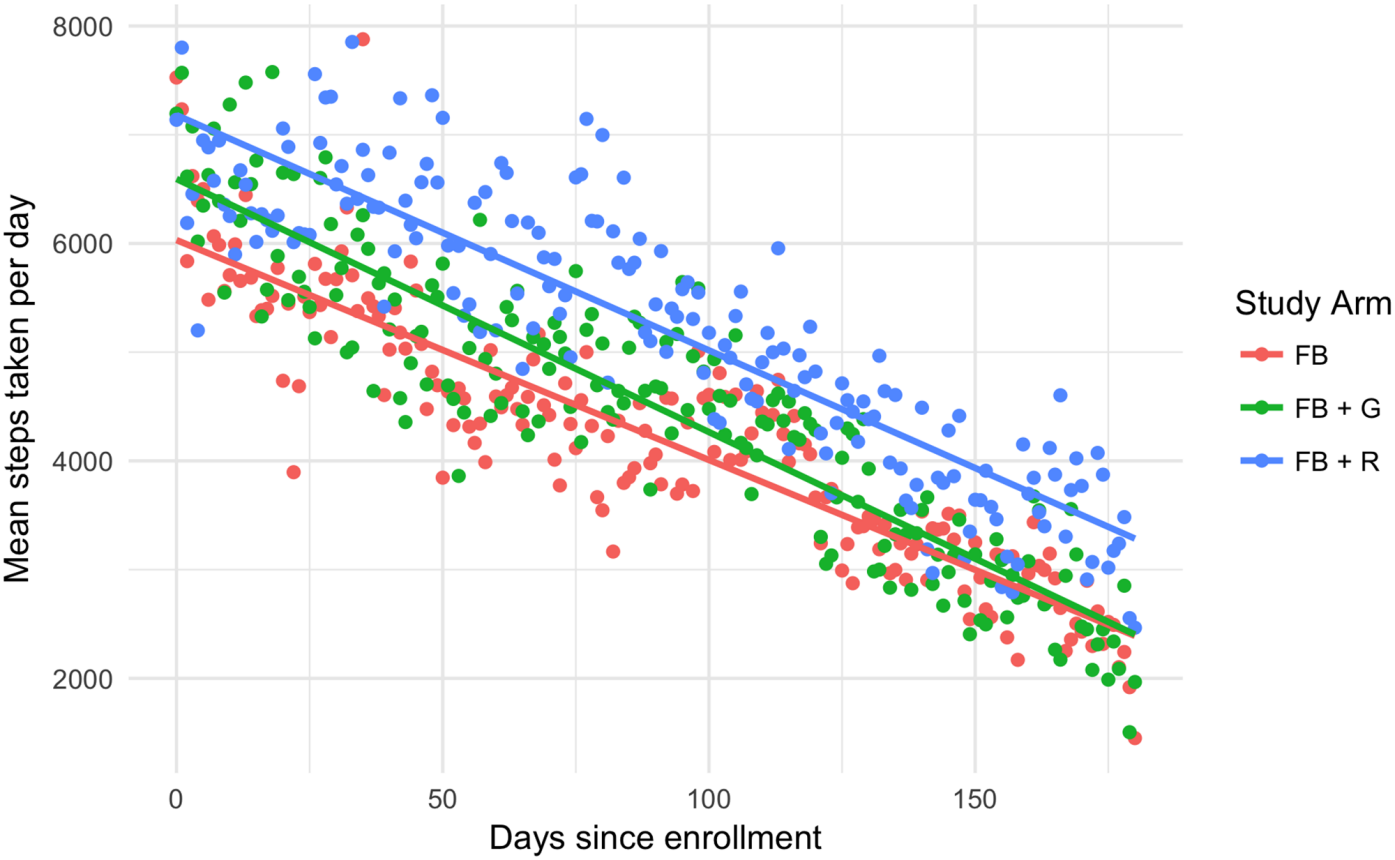
Steps taken per day by group (FB: Fitbit only, FB + R: Reminders, FB + G: Goal-setting).

Of the 28,840 possible person-days, 23,349 (80.96%) had any steps recorded and 15,593 (54.07%) had at least 20 minutes of movement recorded. The number of steps taken, on average, in each group over time is shown in Fig 2. Note that subjects are aligned by enrollment date, but enrolment occurred over a 10-month period.

**Fig 3:**
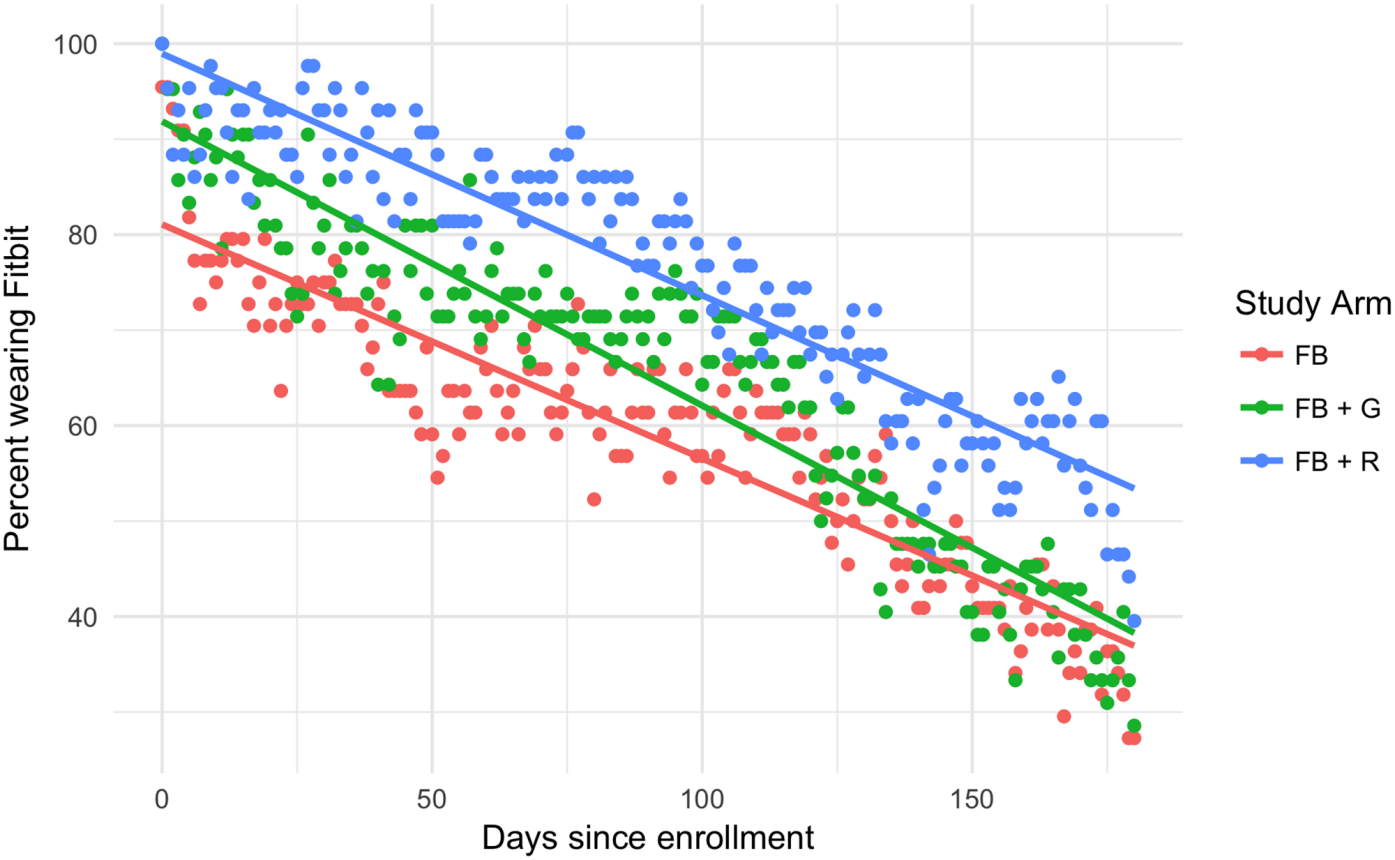
Fitbit use over time and by group.

The Fitbit-only group had the highest average daily steps with 7123 (std dev: 4287). The goal-setting group had fewer average steps than the Fitbit group, but slightly more than the reminders group, with 6909 (std dev: 3748). The reminders group had the fewest average daily steps with 6854 (std dev: 3949). None of these groups were statistically, significantly different from any other. Compliance rates varied considerably between the groups and across time. In general, the Fitbit-only group was the least compliant and the reminders group was most compliant. Compliance rates by group and day are shown in Fig 3. Overall, the reminders group was 17.2 percentage points more likely to wear their Fitbit than the Fitbit-only group (95% CI: 4.8 to 29.4). The goal-setting group was also more likely to wear their Fitbits compared to the Fitbit-only group (+6.1 percentage points, 95% CI: -5.2% to 17.9%), and the difference between the reminders and goal-setting groups was 11.1 percentage points (95% CI: -0.8 to 24.8%). These two results were not statistically significant. Average BMI did not change over the course of the study for any group: the mean BMI was 37.12 at the beginning of the study and 37.16 at the end.

Results of the regression for group membership are shown in Table 2. There was no significant effect of the intervention when comparing goal-setting to either Fitbit only (95% CI: -1,229 to 813) or reminders (95% CI: -846 to 1,135). All groups exhibit negative slopes. We believe that the Fitbit caused steps to increase initially, and the results presented here represent regression to the mean. Each day, activity fell by an average of 6.2 (95% CI: -8.4 to -3.9) steps. This decrease was mitigated by the reminders, and to a lesser extent, the goal setting. The decay among the reminders group is less (3.4 steps/day, 95% CI: -0.3 to 5.2) than the Fitbit-only group. The goal-setting group also had a lower decay (2.5 steps/day, 95% CI 0.8 to 6.0).

**Table 2:** Linear mixed model of daily steps.

Effect	Estimate	95% Confidence Interval	
		Lower Bound	Upper Bound
Intercept	6,713.8	5,965.2	7,473.1
Relative Date	-6.2	-8.4	-3.9
Reminders vs Fitbit Only	-342.8	-1,347.3	664.8
Goals vs Fitbit Only	-182.1	-1,229.1	812.7
Relative Date * Reminders	3.4	-0.3	5.2
Relative Date * Goals	2.5	0.8	6.0

In this model, the outcome variable is daily step count, and 129 participants and 15,593 person-days were included. Covariates include dummy variables for group membership, the number of days since enrollment (relative date), and group membership interacted with relative date. Estimates are adjusted for month of observation.

Estimates for the effect of the number of goals set are shown in Table 3. The effect of setting a goal is associated with a significant increase in the number of steps taken in a day by an average of 791 (95% CI: 361 to 1,223) steps, and this effect does not change over time: when setting a goal is interacted with the number of days since enrollment, the coefficient estimate is not statistically significant. Additionally, setting more goals did not lead to increases in steps: the coefficient on the number of set goals was non-significant and negative. Of the 42 participants in the goal group, 36 submitted at least one goal. The mean number of goals set was 116.5 (median = 138) with an inner quartile range of 77–165 out of the 179 possible goals. The mean goal was 6,035 steps (median = 6,035) with an inner quartile range of 4,000 to 9,000 steps. Goals were set for 3,490 of the 4,953 (70.4%) subject-days in our study.

**Table 3:** Linear mixed model of daily steps and goal setting.

Effect	Estimate	95% Confidence Interval	
		Lower Bound	Upper Bound
Intercept	7,002.2	5,724.1	8,278.3
Relative Date	-9.1	-13.3	-4.9
Set a Goal	790.5	361.2	1,222.6
Number of Goals Set	-5.6	-15.3	4.1
Set a Goal * Relative Date	3.7	-0.2	7.7

In this model, the outcome variable is daily step count, and only the members of the Fitbit+Goals group were included (42 participants and 4,953 person-days). Covariates included the number of days since enrollment (relative date), whether or not the participant had set at least one goal (set a goal), number of goals set by each participant, and an interaction between the relative date and whether or not the participant had set at least one goal. Estimates are adjusted for month of observation.

## Discussion

Our results show a substantial drop-off in the number of steps during the study, which we believe represents a rapid return to baseline levels of physical activity following an initial increase. Neither daily reminders to wear Fitbits nor daily goal-setting messages increased activity levels above those observed from providing subjects Fitbits alone. However, in our population of subjects with obesity, pre-diabetes or diabetes, our two interventions did appear to increase engagement. Specifically, reminder messages and goal setting were associated with increased Fitbit wear time and data collection (i.e., better Fitbit compliance).

In addition, among the goal-setting group, on days when goals were set, an average increase of 791 steps/day occurred. However, we are unable to control for the effect that the subject, at the time of goal setting, has the ability to estimate their step counts for the day and can opt-out of setting a goal. Because subjects in the goal-setting group did not have more steps per day on average than the other two groups, the true effect of goal setting is likely to be smaller after accounting for subjects’ private information.

From our own preliminary work, we knew that compliance (i.e., remembering to wear the Fitbit) poses a significant problem. Indeed, another study found that Fitbit use declined to less than 10% after incentives to wear the Fitbit ended. [22] Thus, in the reminders group, we gave a gentle nudge to improve compliance. We also wanted to test whether this would translate to more physical activity. Subjects in the reminder study arm wore their Fitbits on 25% more days than the Fitbit-only group. Thus, simple reminders can lead to meaningful increases in wear time and the data-capture rate. In addition, as new opportunities for providers to capture patient data outside of traditional healthcare settings arise, adherence to data collection methods and devices will be important. Indeed, text-message reminders have been shown to be effective amplifiers of adherence in other health contexts, [23] and text messaging is an inexpensive intervention. A study in Singapore found that cash incentives also led to greater Fitbit wear, but their incentives totaled an average of S$620 (US$437) per subject. [22]

The Fitbit itself provides activity feedback. However, this feedback may be ignored over time. We were interested in testing whether asking subjects to not only pay attention to their activity levels, but also to actively respond to their activity levels would increase engagement and/or activity. Activity did increase, but engagement did not. Although setting at least one goal was associated with increased steps, the goal setting group was less compliant than the reminders group, though not significantly so. In addition, setting more goals did not lead to further step increases. We believe that these results occurred because of “message fatigue”: having to actively respond to a daily text message became tiresome over time. In the reminders group, text messages were only sent when the subject recorded zero steps the previous day. Because these messages were not sent every day, subjects may have been less likely to ignore them.

Our automated two-way text-messaging platform opens a low-cost and scalable channel for delivering health-behavior-change interventions tied to an individual’s own performance. The potential for our platform should not be overlooked. By connecting the text messages to the individual’s Fitbit measured performance, we are able to send timely, meaningful and personalized messages designed to encourage physical activity to large populations. In the future, we intend to build on this work by further refining our platform to include theory-based behavioral elements for promoting physical activity. While we did provide subjects reminders to wear their Fitbits and to set daily goals, we did not provide subjects with individualized feedback based on progress towards their goals. Consistent with habit formation theories, it is important to reward individuals immediately following achievement of a goal [24]. Because the system integrates with Fitbit data and syncs in near-real-time, we could send users rewarding messages immediately after completing their goals.

Our study has limitations. First, we could not measure activity for subjects when they did not wear their Fitbits, so especially for the Fitbit-only group, the average number of steps could be inaccurate as fewer than 60% of subjects were wearing their Fitbits by the end of the study. However, it should be noted that our Fitbit-based approach for measuring physical activity yielded far more physical activity data when compared to traditional physical activity interventions that measure brief windows of activity at specific time points (i.e., baseline, 6 months). For this reason, we feel confident that we accurately assessed participants’ typical activity behaviors over the course of the study. Second, we used Fitbit Zips, which while relatively accurate, [25] are clip-on devices that may be more easily forgotten than wrist-based accelerometers. On the other hand, Fitbit Zips are powered by a watch battery while wrist-based accelerometers must be recharged every few days.

## Conclusions

Despite these limitations, we found that in a population that volunteered for a physical-activity study, individualized reminders to wear the Fitbit and elicit personal step goals did not lead to increases in daily steps. Although daily steps were higher on days when goals were set, this did not lead to higher average steps for the goal-setting group. This study demonstrates that new, more effective interventions for increasing activity in patients with pre-diabetes and diabetes remain a critical need. However, our intervention did greatly improve engagement and data collection, important goals for improving activity surveillance.

## Supporting information

S1 Dataset Model data. (CSV)

S2 Dataset Clinical data. (CSV)

S1 Text Data dictionary. (DOCX)
