# Supplementary material for: The effect of automated text messaging and goal setting on pedometer adherence and physical activity in patients with diabetes: A randomized controlled trial
Source: PLoS One. 2018 May 2;13(5):e0195797. doi: 10.1371/journal.pone.0195797 (PMC5931450; doi:10.1371/journal.pone.0195797)
Supplement: S1 Text — (DOCX) [file pone.0195797.s003.docx]

The file S1_Dataset_Model.csv contains

link | relative_date | steps | arm | month | minutes

link is variable for linking the two data sets. It is a randomly generated 3-digit number.

relative_date is the number of days since the subject started the study for the steps described in that row

steps is the total number of steps taken on a given day

arm is whether the subject was in FB, FB+G or FB+R

month is the month of year (used in our model to adjust for seasonal variances in activity)

minutes is the number of minutes of activity on relative_date. In our analysis, we required that days have at least 20 minutes of activity but I did not apply that filter to this data set in case someone wants to explore the effect of that rule on our results.

The file S2_Dataset_Clinical_Data.csv contains the clinical variables at enrollment, visits 2 and 3. The two datasets can be linked by the link variable.
